# Cerebral Venous Thrombosis as Rare Presentation of Herpes Simplex Virus Encephalitis

**DOI:** 10.1155/2019/7835420

**Published:** 2019-01-17

**Authors:** José Leite, Ana Ribeiro, Diana Gonçalves, João Sargento-Freitas, Luís Trindade, Victor Duque

**Affiliations:** ^1^Department of Internal Medicine, Coimbra University Hospital Centre, Coimbra, Portugal; ^2^Department of Infectious Diseases, Coimbra University Hospital Centre, Coimbra, Portugal; ^3^Department of Neurology, Coimbra University Hospital Centre, Coimbra, Portugal

## Abstract

Herpes simplex virus 1 is a prevalent neurotropic pathogen that infects and establishes latency in peripheral sensory neurons. It can migrate into the central nervous system and cause encephalitis. The association between herpes simplex virus encephalitis and cerebral venous thrombosis is rare, with a very limited number of case reports described in the literature, despite the recognized thrombogenic effects of the virus. A 44-year-old man was brought to the emergency department with generalized tonic-clonic seizures requiring sedation and ventilation to control it. Initial brain computed tomography revealed cortical and subcortical edema on the left frontal lobe, and a subsequent contrast-enhanced exam showed absence of venous flow over the anterior half of the superior sagittal sinus. Cerebrospinal fluid polymerase chain reaction was positive for herpes simplex virus type 1, and the patient was started on acyclovir and anticoagulation, with clinical improvement. Acyclovir administration was maintained for 14 days and oral anticoagulation for one year, with no recurrence of thrombotic events or other complications. A well-timed treatment has a validated prognostic impact on herpes simplex encephalitis, making early recognition of its clinical aspects of main importance.

## 1. Introduction

Herpes simplex virus 1 (HSV-1) infection is common, with a seropositivity prevalence greater than 60% worldwide [1, 2]. HSV-1 is a neurotropic pathogen that infects and establishes latency in peripheral sensory neurons, but it can migrate into the central nervous system with potentially devastating results. Herpes simplex virus encephalitis (HSE) is an atypical presentation of the infection, with significant mortality and morbidity if not promptly recognized and adequately managed [2]. Despite the prothrombotic effects of the virus [3], the association between HSE and cerebral venous thrombosis (CVT) is extremely rare, with, to the best of our knowledge, only three cases described in the literature [4–6].

## 2. Case Presentation

A 44-year-old white man, with a previous history of gouty arthritis and type 2 diabetes diagnosed two years earlier, was admitted to the emergency department for a reported episode of generalized tonic-clonic seizure at home, lasting 2 minutes. The patient had no recent history of fever or flu-like symptoms but reported a moderate occipital headache in the previous four days. On the initial clinical examination, the patient was lucid and oriented, hemodynamically stable, and with fever (auricular temperature of 38°C). A thorough neurological examination revealed neither meningism signs nor any focal neurological deficit. Fundoscopic examination was normal. Apart from evidence of tongue biting, he had no visible oral or genital vesicular lesions or any skin rash. There were no palpable lymph nodes.

During the observation period in the emergency room, several convulsive episodes were observed, with postcritical agitation and disorientation, requiring sedation with propofol and intubation for airway protection.

**Figure 1:**
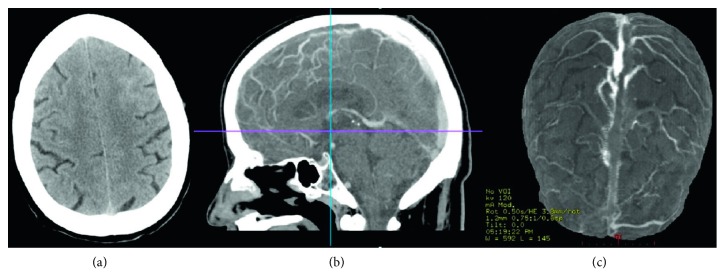
Brain computed tomography images. (a) Initial CT scan reveals loss of cortical-subcortical discrimination in the left frontal lobe and a local linear hyperdensity indicating subarachnoid haemorrhage. (b) CT venography: sagittal cut showing absence of permeability of the anterior two-thirds of the superior longitudinal sinus consistent with thrombosis. (c) 3D reconstruction of the CT venography.

A brain computed tomography (CT) scan was performed, revealing cortical and subcortical edema of the left anterior frontal region and a local linear hyperdensity suggestive of a discrete subarachnoid haemorrhage. A cerebral CT venography revealed venous thrombosis in the anterior two-thirds of the superior longitudinal sinus (Figure 1).

The patient had no prior personal or family history of epilepsy or thrombotic events. There was no history of cancer. His long-term medication was metformin 700 mg and allopurinol 300 mg once a day. He had good metabolic control of type 2 diabetes with a hemoglobin A1c count of 6.2% and no evidence of end-organ damage. Uric acid was in the normal range.

Complete blood count and renal and hepatic function were normal. He had a normal leukocyte count and a red cell distribution width of 13.9%. Inflammatory markers were slightly elevated, the erythrocyte sedimentation rate was 43 mm/h, and the C-reactive protein was 233.3 nmol/L (normal <4.76 nmol/L).

Two sets of blood cultures were collected but had no bacterial growth after 5 days of incubation. An anteroposterior view of a chest X-ray was obtained and showed no evidence of opacities or consolidations. His electrocardiogram had a normal sinus rhythm.

The cytochemical study of cerebrospinal fluid (CSF) revealed 2 leucocytes/mm^3^, a protein level of 230 mg/L, and a glucose level of 10 mmol/L (serum level of 14 mmol/L). The CSF multiplex polymerase chain reaction was positive for HSV-1 and negative for all the other microorganisms tested (HSV-2, enterovirus, varicella zoster virus, human herpes virus 6, cytomegalovirus, *N. meningitidis*, *H. influenzae*, *E. coli* K1, *S. agalactiae*, *L. monocytogenes*, and *C. neoformans*). Serology for HSV was IgG positive and IgM negative and negative for both HIV and syphilis.

The autoimmune study (antinuclear antibodies, anticardiolipin antibodies, and lupus anticoagulant) was negative or normal. Complement C3 and C4 test and homocysteine were normal. Protein C, protein S, and antithrombin III levels were normal. The molecular study of genetic thrombophilias was negative for prothrombin 20210G>A mutation but revealed heterozygosity for factor V Leiden (mutation 1691G>A).

The diagnosis of CVT secondary to HSV-1 infection in a patient with heterozygosity for factor V Leiden was made. The treatment was started with enoxaparin 1 mg/kg twice daily, levetiracetam 1500 mg bid, and acyclovir 800 mg tid for 14 days. The patient had a positive clinical evolution during hospitalization, with no more seizures and no neurological deficits at discharge. The selected oral anticoagulant was rivaroxaban 20 mg id, maintained for 12 months, without any new convulsive or thromboembolic episodes registered.

## 3. Discussion

HSV-1 is estimated to affect more than 60% of world population and is the main etiological agent of sporadic encephalitis [1, 2]. The diagnosis is suggested by altered mental status, with or without fever, associated with laboratory and imaging evidence of inflammation of the central nervous system. A clinical picture of headache, seizures, and focal neurological deficits should make one think of herpetic encephalitis. CSF may usually present moderate lymphocytic pleocytosis (10–200 lymphocytes/mm^3^). Brain CT has low sensitivity and diagnostic specificity, with brain magnetic resonance imaging (MRI) being the examination of choice for the diagnosis of encephalitis, although it is not always available in an emergency room. The definitive diagnosis is made by a positive multiplex PCR for HSV in the CSF. This test has high sensitivity (96%) and specificity (99%) and supplanted virus cultures as the test of choice for definitive diagnosis [2]. HSV serology is of little interest in the acute setting [2].

If clinical suspicion is high, treatment should be initiated as soon as possible. The early introduction of acyclovir is the modifiable factor with the most significant impact on prognosis [2]. Empirical treatment is indicated for suspected cases, given the potential benefit compared to the relative innocuity of the drug. The delay in therapy initiation is one of the factors responsible for poor prognosis, along with age and altered mental status at presentation [2]. A dose of 30 mg/kg/day (10 mg/kg tid) is recommended for 14 to 21 days, depending on the clinical response. Untreated HSV-1 encephalitis typically had a high mortality rate (about 70%) that significantly decreased to 5–15% with the advent of acyclovir [2]. Although it seems to improve the outcome in animal models, the role of corticosteroids in the treatment of HSE is not yet well established, and their use is reserved for situations in which there is significant brain edema and mass effect [2].

Recurrence is uncommon in adults and is generally less severe than the initial episode. It is, however, more related to immune-mediated phenomena than to virus activity itself [2].

The most frequent complications of HSE are seizures and brain edema. HSE may rarely present or evolve with intracranial bleeding [7], most often in the form of scattered petechial haemorrhages. This complication may occur even after the initiation of treatment, and the lack of clinical improvement or sudden clinical worsening should raise suspicion of a vascular complication.

Endothelial cell (EC) injury is a common feature of viral infections and can alter hemostasis.

Herpesviridae infections, particularly, inhibit anticoagulant functions and induce a procoagulant phenotype. However, if on one hand the vasculopathy associated with the varicella zoster virus is well known, having a wide clinical range that goes from transient ischemic attacks and cerebral and subarachnoid haemorrhages to spinal-cord infarction [8], on the other hand the vascular involvement in herpes simplex infections is not so conspicuous.

There are several mechanisms, studied in vitro, that were proposed as contributing factors to increased thrombotic risk in HSV infections [3]. By infecting EC, HSV-1 and HSV-2 reduce heparan sulfate proteoglycan synthesis and expression, therefore preventing antithrombin III binding to its surface. The expression of thrombomodulin is also reduced, affecting protein C activation.

Changes in the EC membrane lead to enhanced thrombin generation, increasing platelet binding and decreasing prostacyclin synthesis. Another mechanism proposed is that HSV infection increases endothelial binding sites for inflammatory cells and platelets, with subsequent release of inflammatory cytokines and potentiation of a prothrombotic EC surface.

Although the prothrombotic effects of HSV-1 are recognized and debated in the literature, CVT is an extremely rare event in a proven association with herpetic encephalitis [3]. To our knowledge, there are only another three cases reported in the literature (Table 1). All patients were aged between 30 and 50 years and had a similar clinical presentation, with seizures and headaches being the predominant symptoms. In three of the four cases, the diagnosis of HSE and CVT was made simultaneously and in one case [6], there was a clinical worsening during HSE treatment that led to CVT diagnosis. In two of the cases, there was an identifiable risk factor for venous thromboembolism. Our patient is the only one that received a direct anticoagulant as maintenance treatment after the acute phase. There were no neurological deficits at hospital discharge in any case nor were any recurrences of the symptoms reported.

CVT has a prothrombotic risk factor identified in 85% of the cases [9], either genetic or acquired. Its clinical manifestations are vast, from headaches to nausea and vomiting or signs of intracranial hypertension, focal neurological syndromes, and encephalopathy. The diagnosis is usually made by contrast-enhanced CT or MRI. The therapeutic approach should be anticoagulation, even in the presence of intracerebral haemorrhage [9–11]. The initiation of anticoagulation should be done with low-molecular weight heparin or conventional heparin until the patient is stable and then switching to oral anticoagulation with warfarin that should be continued for 3 months (in the presence of transient risk factors) or 6–12 months (in the presence of nonmodifiable risk factors). Indefinite anticoagulation should be considered in the presence of major risk factors, namely, severe thrombophilia (antithrombin deficiency, protein C or S, antiphospholipid syndrome, and homozygosity for factor V Leiden) [11]. Factor V Leiden is the genetic risk factor most often found in patients with venous thrombosis. Heterozygosity has a prevalence of 3–8% in the European population. The risk of cerebral venous thrombosis is 3–5 times normal in heterozygotes [12].

The role of direct anticoagulants in the anticoagulation of cerebral thrombotic events is still not consensual. Although its therapeutic role in pulmonary and lower limb venous thrombosis is already widely supported and recommended, cerebral venous thrombosis relies on a considerably different pathophysiological basis, and its use for this purpose should be considered off-label [10, 13]. The available literature relies essentially on a small series of clinical cases [11, 14], and therefore the quality of evidence is still considered very low. There is no evidence of inferiority in relation to warfarin, either in terms of efficacy or safety [12, 13], but its use requires close counseling and follow-up. In the described clinical case, the authors consider that the association of a genetic risk factor with HSV-1 central nervous system infection caused the thrombotic event. Anticoagulation with rivaroxaban was chosen after a multidisciplinary team discussion on the available therapeutic options.

## 4. Conclusion

HSV-1 encephalitis in association with CVT is a rare event with very few cases reported.

The management of both events requires early therapeutic measures with great impact on the prognosis.

The role of direct oral anticoagulants as a therapeutic arsenal for thrombotic events is expanding, but its use in the treatment of CVT requires more scientific support and should be adapted to each clinical case individually.

## Figures and Tables

**Table 1:** Comparison between cases of HSV encephalitis associated with CVT reported in the literature.

Case number		Year of publication	Patient's age	Patient's sex	HSV clinical presentation	Diagnosis of CVT	Risk factors identified for venous thromboembolism	CVT treatment	Outcome
Case 1 [4]	2005		48	Male	Seizures, high fever, history of headaches, and flu-like symptoms	Simultaneous	None	LMWH acute phase followed by warfarin for 6 months	Favourable, no deficits
Case 2 [5]	2012		30	Female	Seizures, high fever, confusion, and headaches	Simultaneous	Pregnancy	LMWH until delivery	Favourable, no deficits
Case 3 [6]	2018		31	Male	Seizures, headache, and photofobia	Day 6 of treatment: worsening headache, upper limb weakness	None	Intravenous heparin acute phase followed by warfarin for 1 year	Favourable, no deficits
Our case	—		44	Male	Seizures, low fever, and history of headaches	Simultaneous	Factor V Leiden heterozygosity	LMWH acute phase followed by rivaroxaban 1 year	Favourable, no deficits

LMWH: low-molecular-weight heparin.
